# Altered mental status in “Guillain‐Barré syndrome” –a noteworthy clinical clue

**DOI:** 10.1002/acn3.51226

**Published:** 2020-11-02

**Authors:** Eoin Mulroy, Neil E. Anderson

**Affiliations:** ^1^ UCL Queen Square Institute of Neurology London UK; ^2^ Auckland District Health Board Auckland New Zealand

## Abstract

Guillain‐Barré syndrome (GBS) is widely regarded as a “pure” peripheral nervous system disorder. However, this simplistic interpretation belies the fact that central nervous system involvement, often manifesting as derangements in mental status can occur as a complication of the “pure” form of the disorder, as part of GBS variants, as well as in a number of mimic disorders. Despite being common in clinical practice, there is no guidance in the literature as to how to approach such scenarios. Herein, we detail our approach to these cases.

## Introduction

Guillain‐Barré syndrome (GBS) is the commonest cause of acute flaccid quadriparesis in the developed world.^1^ The classic syndrome is one of acute inflammatory demyelinating polyradiculoneuropathy characterized by the rapid onset of areflexic weakness, usually affecting the limbs both proximally and distally, and by definition reaching nadir within 4 weeks.^2^ In a quarter of cases, respiratory function becomes compromised, requiring intubation and ventilation.^3^ Other characteristic features include autonomic instability, back pain secondary to radicular inflammation, and sensory symptoms without striking sensory signs.^1^ Diagnosis remains clinical, though features such as cerebrospinal fluid albuminocytologic dissociation and neurophysiologic evidence of demyelinating polyradiculoneuropathy (both often absent early in the disease) can be helpful. Numerous forme frustes of GBS have also been defined, including axonal variants, pharyngeal‐brachial variant, isolated bilateral facial nerve palsy, and the anti‐Gq1b antibody spectrum of disorders.^2^

Central nervous system dysfunction is classically considered not to be a part of GBS. However, in our experience, it is not uncommon for alterations in mental status to be present in patients who appear to otherwise have GBS. This has often left us perplexed, questioning our original diagnosis and uncomfortably adrift in our approach. After many hours of scouring the literature in search of guidance, we noted a dearth of information on this topic, prompting us to write this review.

## General Approach

**Figure 1:**
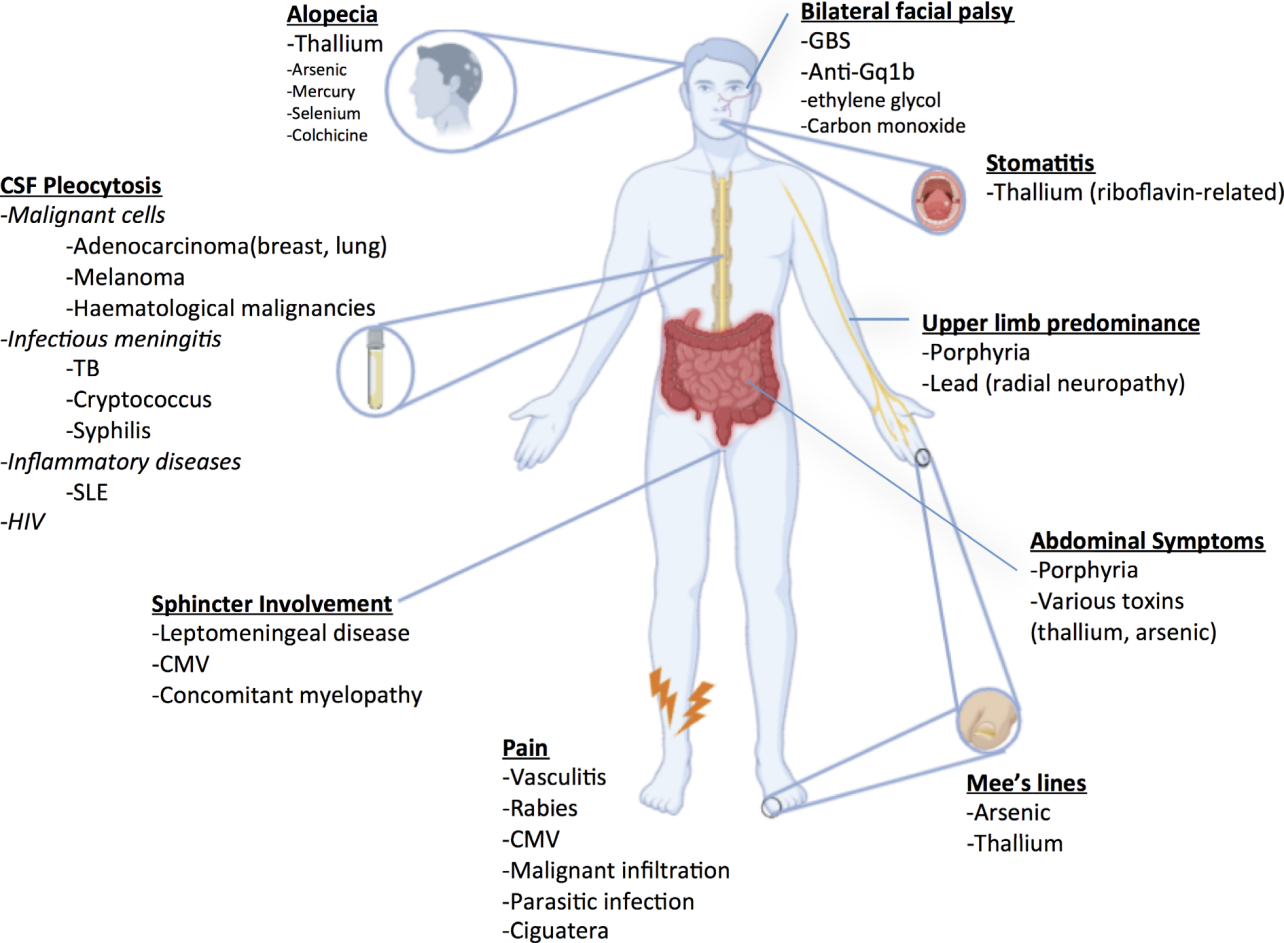
Clinical clues suggesting GBS mimics with altered mental status (GBS + CNS). CSF, cerebrospinal fluid; CMV, cytomegalovirus; HIV, human immunodeficiency virus; SLE, systemic lupus erythematosus; TB, Tuberculosis.

**Figure 2:**
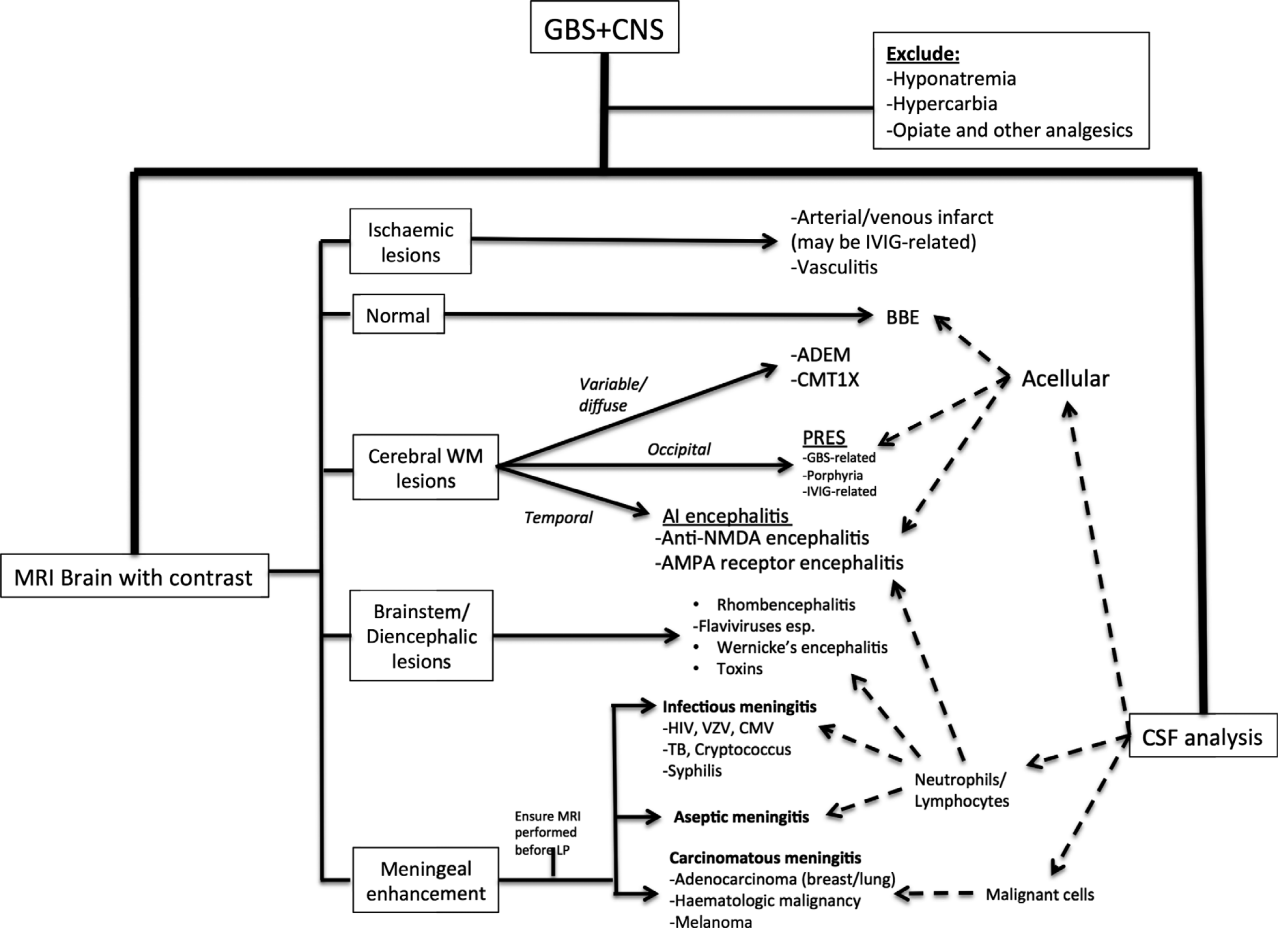
Clinical algorithm for approaching cases of suspected GBS + CNS. ADEM, acute disseminated encephalomyelitis; AI, autoimmune; AMPA, α‐amino‐3‐hydroxy‐5‐methyl‐4‐isoxazolepropionic acid; BBE, Bickerstaff brainstem encephalitis; CMT 1X, Charcot‐Marie‐Tooth type 1X; CSF, cerebrospinal fluid; CMV, cytomegalovirus; HIV, human immunodeficiency virus; IVIG, intravenous immunoglobulin; LP, lumbar puncture; MRI, magnetic resonance imaging; NMDA, N‐Methyl‐ d‐aspartate; PRES, posterior reversible encephalopathy syndrome; TB, Tuberculosis; VZV, varicella zoster virus; WM, white matter.

An altered sensorium in a patient who appears to have GBS deserves more than just flippant acknowledgement. Indeed, while clouding of consciousness or neuropsychiatric symptoms can be intrinsic features of GBS, they may also portend significant complications of the disease, treatment‐related adverse events, or importantly, may suggest an alternative diagnosis. This review presents clinicians with a pragmatic, practical approach to assessing patients with suspected GBS who exhibit unanticipated CNS signs, herein termed “GBS + CNS” (see Figure 1). As this is intended as a clinical review, the discussion has purposefully been structured in terms of the questions which clinicians should ask themselves as they approach such cases. We also present a decision tree (Figure 2), to which clinicians may refer in their diagnostic process.

## Could the Observed Mental Status Changes be Part of the Guillain‐Barré Syndrome?

Broadly speaking, mental status changes as part of a “true” GBS illness can comprise either:

1. Intrinsic neuropsychiatric manifestations of the disorder, or
2. CNS dysfunction resulting from disease‐related autonomic or metabolic derangements.

### Intrinsic neuropsychiatric manifestations of GBS

Clinical descriptions of GBS primarily focus on peripheral nervous involvement, and rightly so. Nevertheless, it must be borne in mind that CNS dysfunction, ranging from mild neuropsychiatric symptoms (depression, anxiety)^4^ to deep unconsciousness (mainly in children, where GBS may coexist with acute disseminated encephalomyelitis‐ADEM) is recognized as part of the GBS spectrum.^5^, ^6^, ^7^ In fact, a systematic study of critically ill adult patients with GBS found that almost one third had mental status abnormalities.^8^ Encephalopathy was disproportionately common in patients with higher CSF protein levels and autonomic dysfunction, had peak incidence at one week following disease onset, and tended to persist for a further week. Hypnagogic or hypnopompic vivid dreams charged in emotional content, illusions (visual, tactile, auditory, and illusions of body tilt), visual and tactile hallucinations as well as delusions (usually paranoid) were all examples of mental status abnormalities reported in this study as intrinsic GBS‐related features.^8^

Though GBS‐related neuropsychiatric alterations remain a diagnosis of exclusion coming at the end of any decision tree once every reasonable effort has be made to rule out other causes, we thought it important to highlight its existence right from the outset in this review.

### Metabolic Disturbances associated with GBS

Metabolic disturbances are common among hospitalized patients regardless of underlying diagnosis. Specific to GBS however, symptomatic hyponatremia secondary to the syndrome of inappropriate antidiuretic hormone secretion (SIADH), CO2 narcosis (as mentioned before, 25% of patients require ventilation as a result of weakness‐related hypoventilation) and decubitus‐related infections should all be actively searched for.

SIADH is particularly common, affecting roughly 50% of GBS patients. Its pathogenesis is incompletely understood, but multiple theories have been proposed. These include increased ADH release secondary to hypothalamic damage or immune mechanisms,^9^, ^10^ resetting of hypothalamic osmoreceptors^11^ and altered renal tubular sensitivity to ADH.^12^ It is more common in older patients and those with bulbar dysfunction and/or ventilator dependency,^12^ and manifests clinically with altered mental status, seizures and occasionally coma (depending on the rapidity and severity of hyponatremia, and subsequent fluid shifts) and biochemically with euvolemic, hypo‐osmolar hyponatremia. In those receiving IVIG, it should not be confused with IVIG‐related pseudohyponatremia,^13^ an analytic artifact which is easily differentiated from true hyponatremia by the absence of hypo‐osmolality.^14^ Hyponatremia must be managed with care, as overly rapid correction can precipitate osmotic demyelination syndromes, producing permanent, rather than temporary alterations of consciousnesss.^15^

### Autonomic dysfunction

Autonomic dysfunction secondary to demyelination of autonomic nerve fibers is considered a hallmark feature of GBS, being present in at least two thirds of cases.^16^ Blood pressure variability is particularly characteristic, occasionally culminating in either sustained severe systolic hypertension or cardiovascular collapse.^17^

While malignant hypertension may in and of itself produce mental status changes, one condition to be particularly mindful of in GBS + CNS is posterior reversible encephalopathy syndrome (PRES). ***PRES*** is a clinico‐radiologic entity manifesting classically with headache, nausea, vomiting, visual changes, seizures, encephalopathy, and focal neurologic deficits alongside radiologic evidence of vasogenic edema predominating in posterior brain regions.^18^ Its pathophysiologic basis is incompletely understood but rapidly developing hypertension with failure of cerebral autoregulation is thought to be critical. The relative lack of sympathetic innervation of the posterior circulation potentially accounts for preferential involvement of the posterior brain regions. Most cases of GBS‐related PRES have occurred in women aged> 55 years.^19^ Both GBS‐associated dysautonomia and IVIG treatment offer fertile territory for its development.

## Could the GBS Treatment be Responsible?

One should always consider that GBS + CNS could be an iatrogenic phenomenon resulting from either disease‐modifying or symptomatic treatment administered for GBS.

Intravenous immunoglobulin (IVIG) has become a standard of GBS care worldwide, in an attempt to hasten recovery.^20^ Though often perceived as a benign treatment, it is certainly not devoid of adverse effects. The incidence of ***thrombotic events*** is variable (1–16% in the literature), generally occurring within 24 hr of administration of IVIG.^21^ Patients with a history of prior thrombosis, advanced age, high total daily dose, and underlying prothrombotic tendencies or atherosclerosis are at heightened risk. Such thromboses can involve both cerebral arterial and venous systems, and should be considered in GBS + CNS patients who are receiving IVIG, especially if other suggestive signs (either focal neurological deficits or –especially in intracranial venous thromboses‐ papilloedema, headache or seizures) are present.^21^ ***Aseptic meningitis*** affects 0.5–1% of IVIG‐treated patients, generally begins within 48 h of starting the infusion and can rarely lead to mental status alterations, generally alongside fever, headache and meningismus.^22^, ^23^ CSF examination in such cases reveals an aseptic (often neutrophilic) pleocytosis, elevated CSF protein and high opening pressure.^22^ The symptoms are often indistinguishable from viral or bacterial meningitides in the early stages; the condition generally settles spontaneously in 5–7 days.^23^ As mentioned before, IVIG administration can also trigger ***PRES***.

Opiate and nonopiate analgesics (e.g., pregabalin, gabapentin), commonly administered for neuropathic pain in this population, should also make the shortlist of potential culprits for mental status alteration. Additionally, in ventilated patients, one should always consider the effects of sedative medications (or withdrawal therefrom) on mental status.

## Am I Dealing with a GBS Variant (esp. Anti‐Gq1b Ssease)?

The next point to consider is whether the patient could be suffering from a related, but distinct disorder, particularly anti‐Gq1b antibody‐related disease.^24^ Anti‐Gq1b syndromes manifest along a spectrum, ranging from the peripheral nervous system predominant Miller‐Fisher syndrome (typified by the clinical triad of ataxia, areflexia and ophthalmoplegia) through to central involvement in the form of Bickerstaff’s brainstem encephalitis, defined by a combination of external ophthalmoplegia AND either altered level of consciousness –ranging from mild drowsiness to coma‐ OR hyper‐reflexia^25^, ^26^, ^27^ with a multitude of forme frustes including acute ophthalmoparesis and acute ataxic neuropathy in between.^24^, ^28^

Up to 50 percent of patients with Bickerstaff’s brainstem encephalitis have coexisting GBS, but limb weakness generally follows the development of ophthalmoplegia/ataxia rather than being present at the onset.^27^ Nerve conduction studies in these patients frequently demonstrate axonal involvement. Other common symptoms include facial weakness, bulbar dysfunction, blepharoptosis, mydriasis, nystagmus, and proprioceptive deficits; 25–30% may display nonspecific MRI abnormalities, consisting of T2‐weighted hyperintensities in the brainstem, thalamus, cerebellum, or cerebral white matter.^27^

## Am I Wrong, and This Actually is Not GBS?

Having considered disease‐related CNS involvement/complications, side‐effects of therapy and disease variants, one should ponder whether mental status changes actually suggest the presence of a GBS mimic. Indeed, numerous disorders, some of which are eminently treatable, manifest with the combination of acute paresis alongside CNS dysfunction.

While an extensive list of such disorders is given in Tables 1, 2, 3, 4, 5, these inventories are often unhelpful in the clinical setting. We find the mnemonic MIND (Metabolic + Malignancy, Infectious/Immune/Inherited, Nutritional, Drugs/toxins) useful to classify these remaining diseases, the most pertinent of which we discuss below.

**Table 1:** Causes of GBS + CNS in the presence of an initial “correct” GBS diagnosis.

Condition	Clinical Clues	Additional Investigations
1. Initial Diagnosis Correct		
Intrinsic feature of GBS illness	− week 1‐2‐hypnagogic/hypnopompic dreaming/misperceptions − hallucinations + delusions	MRI Brain normal EMG/NCS may be suggestive CSF albuminocytologic dissociation
Intrinsic feature of GBS variant		
Anti‐Gq1b disease	− Ataxia − Areflexia − Ophthalmoplegia − +/‐ GBS‐like weakness	Serum anti‐Gq1b antibody positivity Brain MRI usually normal
Complications of disease		
*Metabolic derangement*		
Hyponatremia (SIADH)	− Non‐specific: ‐ Headache ‐ Nausea ‐ Vomiting ‐ Weakness ‐ Confusion ‐ Seizures	Serum sodium, Serum Osmolality, Urine Osmolality.
Hypercarbia (respiratory muscle weakness)	− Flushing − Bounding pulses − Drowsiness/confusion	Arterial blood gas ‐ CO2 levels
Infections	Signs of pulmonary, urinary or skin infection especially	Elevated WCC, CRP; Abnormal CXR or urinalysis
*Autonomic instability*		
Posterior reversible encephalopathy syndrome (PRES)	− Headache − Seizure − Visual disturbance − Focal neurologic signs	T2 and FLAIR hyperintensities predominantly in posterior brain regions
Complication of treatment		
*IVIG‐relatesd*		
Hypercoagulability(also rarely encountered with plasmapheresis)	Focal neurological deficits(arterial/venous)+/‐seizures, headache, papilloedema(venous)	MRI Brain: Diffusion restriction; vascular imaging help define cause and extent of thrombosis
PRES	as above	as above
Aseptic meningitis	− Headache ‐ ‐Fever ‐ Meningism	Culture‐negative CSF pleocytosis
*Medications* − Opioids − Gabapentin/Pregabalin − Tricyclic antidepressants − Anaesthetic/sedative drugs	− Opioid: pinpoint pupils, depressed respiratory rate	Medication administration review

CNS, Central Nervous System; CRP, c‐reactive protein; CO2, carbon dioxide; CSF, cerebrospinal fluid; CXR, chest X‐ray; EMG, Electromyography; FLAIR, fluid‐attenuated inversion recovery; GBS, Guillain‐Barré Syndrome; IVIG, intravenous immunoglobulin; MRI, magnetic resonance imaging; NCS, nerve conduction studies; PRES, posterior reversible encephalopathy syndrome; SIADH, syndrome of inappropriate ADH secretion; WCC, white cell count

**Table 2:** Initial diagnosis incorrect‐ consider “MIND” disorders: M = Metabolic/Malignancy.

Condition	Clinical Clues	Additional Investigations
Metabolic/Malignant		
*Metabolic*		
Porphyria	− Psychiatric symptoms − Abdominal pain − Seizures − Autonomic instability ++ +/‐ triggers: Surgery, infection, enzyme‐inducing medications	Elevated urinary aminolevulinic acid and porphobilinogen during attack
Thyrotoxicosis	− Lower‐limb predominant acute polyradiculonauropathy –“Basedow paraplegia” (rare manifestation) − CNS signs: anxiety, psychosis, mood disorder, confusion − Systemic signs: tachycardia, heat intolerance, weight loss, ophthalmopathy	Low serum TSH Elevated serum T3 and free T4
Mitochondrial disorders	PDH complex deficiency especially can present recurrent peripheral weakness in childhood. GBS + CNS occasionally encountered in other mitochondrial disorders	‐Bloods: Lactic acidosis, hyperalaninemia ‐MRI: Symmetrical basal ganglia/brainstem lesions ‐Muscle biopsy ‐Genetic analysis
*Malignant*		
Carcinomatous meningitis	− Asymmetric − Progressive − Spinal and or cranial radiculoneuropathies History of cancer, esp: − Melanoma, adenocarcinoma, hematologic malignancies	CSF: Malignant cells MRI with contrast: Meningeal contrast uptake
Lymphoma (Non‐Hodgkin>>>Hodgkin)	GBS‐like illnesses due to either: 1. Direct neural invasion (neurolymphomatosis, lymphomatous meningitis) or, 2.Immune mechanisms	MRI Brain: generally abnormal‐enhancing mass lesion(s) CSF: cytology, flow cytometry Systemic staging: PET‐CT Biopsy of affected tissue(brain, vitreous) necessary for diagnosis.
Paraneoplastic syndromes^1^ − Anti‐Hu − Anti‐CRMP5	CNS involvement: − Cerebellar syndrome (severe, progressive) − Encephalitis − Opsoclonus‐myoclonus PNS involvement: − Sensory neuronopathy − GBS‐like neuropathy − Dysautonomia − Lambert‐Eaton syndrome	Onco‐neuronal antibody screen Search for primary malignancy

CSF: cerebrospinal fluid; CNS: central nervous system; CT: computed tomography; GBS: Guillain‐Barré Syndrome; MRI: magnetic resonance imaging; PDH: pyruvate dehydrogenase; PET: positron emission tomography; PNS: peripheral nervous system; TSH: thyroid‐stimulating hormone.

^1^ Presentation usually largely dominated by central nervous system involvement. PNS signs rare, but occasionally reported

**Table 3:** Initial diagnosis incorrect‐ consider “MIND” disorders: I = Infections/Immune/Inherited.

Condition	Clinical Clues	Additional Investigations
Infections		
*Enteroviruses* − Poliovirus − EV‐D68 − EV‐71	Anterior horn cell involvement ‐> paralysis +/‐Brainstem encephalitis	RT‐PCR of cerebrospinal fluid
*Flaviviruses* − West Nile Virus − Japanese Encephalitis Virus − Tick‐Borne Encephalitis Virus − St.Louis Encephalitis Virus − Murray Valley Encephalitis Virus − Zika Virus − Dengue Virus	‐Anterior horn cell involvement ‐> asymmetric paralysis (except Zika virus, which produces classic GBS) ‐Encephalitis ‐Travel history important ++	Virus‐specific IgM and IgG antibodies
*Lyssavirus* − Paralytic Rabies	‐Prodromal pain in bitten limb ‐Early fasciculations ‐Rapidly progressive course	RT‐PCR of saliva Rabies antibodies in CSF or serum
*HIV*	‐GBS occurs early ‐CNS signs occur late	HIV p24 antigen, PCR viral load, ELISA or Western blot
*Herpesviruses* *‐CMV* *‐VZV*	CMV: − Significant immunosuppression − Painless visual loss (CMV retinitis) − Hepatitis − CMV radiculitis painful ++ and sphincter‐involving VZV: − Primary/Reactivation − +/‐Rash	Positive CSF PCR for CMV/VZV
*Lyme*	− Spinal and cranial neuritis +/‐ encephalitis (rare) − History of tick bite	CSF and serum Lyme ELISA +/‐ confirmatory Western blot
*Other infections* − *Tuberculosis* − *Syphilis* − *Cryptococcus*	− Polyradiculoneuropathies esp. in immunosuppressed	Serum +/‐ CSF testing for individual pathogens
*Parasites:* − *Angiostrongylus Cantonensis* − *Gnathostoma Spinigerum*	− Southeast Asia + Pacific − Consumption of snails, slugs (Angiostrongylus) or infected fish, seafood (Gnathostoma). − Painful radiculitis+/‐altered mentation − Dermatologic manifestations‐linear dermatitis, migratory panniculitis in Gnathostomiasis	Serum and CSF eosinophilia Specific diagnosis requires ELISA or immunoblot
Immune		
Vasculitis	SLE: − skin (alopecia, oral ulcers, malar rash) − inflammatory arthritis − hematological (anemia, leucopenia, thrombocytopenia) − renal (proteinuria, renal failure) − serosal (pericarditis, pleuritis) EGPA: − asthma − sinusitis − systemic vasculitis	SLE: − +ve antiphospholipid antibodies − Low C3 and C4 − +ve anti‐dsDNA and anti‐Sm antibodies EGPA − hypereosinophilia; pulmonary infiltrates (CXR) − P‐ANCA/MPO‐ANCA positive − Biopsy: eosinophil‐rich granulomatous vasculitis
Neurosarcoidosis	− Cranial neuropathies (facial and optic nerves) − Headache − Spinal cord involvement − Meningitis +/‐ Lung, joint, eye, skin, and lymph node involvement.	CSF pleocytosis CSF ACE levels may be elevated Definite diagnosis: biopsy
Sjogren syndrome	‐F>>M(9:1 ratio) ‐Ocular and oral dryness ‐Musculoskeletal pain and fatigue +/‐pulmonary, renal or biliary tract involvement. Neuro: ‐Sensory neuropathy/neuronopathy	Anti‐SSA and/or anti‐SSB antibodies Schirmer’s test ‐ ocular dryness Minor salivary gland biopsy
Autoimmune encephalitides^1^ *‐AMPA* *‐anti‐NMDA* *‐Anti‐CASPR2*	− Limbic Encephalitis − Movement Disorders − Autonomic Dysfunction +/‐ Neuropathy	Autoimmune encephalitis antibody panel T2/FLAIR hyperintensities in medial temporal lobes CSF pleocytosis
*Inherited*		
CMT 1X	− Sensorimotor demyelinating neuropathy − Transient neurologic episodes: hemiparesis, paraparesis, quadriparesis, dysarthria, ataxia, altered mental state Triggers: infections, exertion, trauma, altitude.	Genetics: *GJB1* pathogenic variants

ACE, angiotensin‐converting enzyme; AMPA, α‐amino‐3‐hydroxy‐5‐methyl‐4‐isoxazolepropionic acid; ANCA, Anti‐neutrophil cytoplasmic antibodies; CASPR2, Contactin‐associated protein‐like 2;CMT 1X, Charcot‐Marie‐Tooth type 1X; CMV, cytomegalovirus; CNS, central nervous system; CSF, cerebrospinal fluid; CXR, chest X‐ray; dsDNA, double‐stranded DNA; EGPA, eosinophilic granulomatosis with polyangiitis; ELISA, enzyme‐linked immuosorbent assay; EV, enterovirus; F, female; FLAIR, fluid‐attenuated inversion recovery; GBS, Guillain‐Barré Syndrome; HIV, human immunodeficiency virus; M, male; MPO, myeloperoxidase; NMDA, N‐Methyl‐ d‐aspartate; RT‐PCR, reverse transcription polymerase chain reaction; SLE, systemic lupus erythematosus; VZV, varicella zoster virus

^1^ Presentation usually largely dominated by central nervous system involvement. PNS signs rare, but occasionally reported

**Table 4:** Initial diagnosis incorrect‐ consider “MIND” disorders: N = nutritional.

Condition	Clinical Clues	Additional Investigations
Nutritional		
Thiamine (Vitamin B1)	− Wernicke's Encephalopathy: Confusion, Ataxia, Ophthalmoplegia − Korsakoff Syndrome: Amnesia, confabulation +/‐Thiamine Neuropathy +/‐Cardiac involvement	Serum thiamine levels (rarely useful‐long turnaround time) − Treat upon suspicion
Vitamin B12^1^	− Subacute combined degeneration of the spinal cord +/‐ neuropathy, cognitive changes(rare)	Low serum vitamin B12 Elevated homocysteine and methylmalonic acid Macrocytic anemia with Hypersegmented neutrophils
Copper^1^	− Similar syndrome to Vitamin B12 deficiency. − At risk: bariatric surgery, excess zinc supplementation	Low serum copper Low serum ceruloplasmin

^1^ Presentation usually largely dominated by peripheral nervous system involvement. CNS signs rare, but occasionally reported

**Table 5:** Initial diagnosis incorrect‐ consider “MIND” disorders: D = drugs/toxins.

Condition	Clinical Clues	Additional Investigations
Drugs/toxins		
Arsenic	− Abdominal symptoms + bloody diarrhea − Weeks 1‐2: neuropathy − Palmo‐plantar desquamation − Mee's lines − Alopecia − CNS signs: encephalopathy, psychosis.	Elevated urine arsenic levels
Thallium	− Painful peripheral neuropathy − Psychosis − Movement disorder − Stomatitis − Alopecia (onset week 2‐6)	Elevated urinary and blood thallium levels
Lead^1^	− Motor‐predominant neuropathy − Upper limbs> Lower limbs, esp. radial nerve − Lead encephalopathy mainly affects children − Abdominal discomfort − Proximal tubular nephropathy	FBC: microcytic anemia with basophilic stippling of RBCs Blood lead levels: increased
Selenium	− Rare:suicide attempts/excessive dietary consumption (Brazil nuts, health supplements) − Irritability, lethargy, confusion, depression − Brittle, discolored nails − Alopecia − “garlicky” breath +/‐Peripheral neuropathy	serum selenium: elevated 24 hr urine selenium excretion: elevated
Botulism^1^	− Descending paralysis: ptosis, diplopia followed by bulbar dysfunction and later limb weakness. − Altered mental status very rare (unless hypoxic/other cause)	Toxin identification: serum, stool, or food sample ‐Mouse bioassays, ELISA, PCR
Snake envenomation^1^	− Rapid descending paralytic syndrome +/‐ autonomic instability − CNS signs rare (may occur as a result of hemorrhage)	Clinical diagnosis‐ descriptions of the causative snake may help identification
Ethylene glycol Diethylene Glycol^2^	− 0‐12 hours: Inebriation, ataxia, stupor, coma, seizures − 12 hrs‐3 days: Cardiopulmonary failure, gap acidosis, renal failure − 5‐14 days: Flaccid areflexic weakness,bulbar weakness, bilateral facial palsy. +/−CNS signs esp. parkinsonism	‐Increased anion gap metabolic acidosis ‐Increased osmolal gap ‐Calcium oxalate crystalluria ‐Elevated serum glycol levels
Toluene^2^	− Glue sniffing. − Headache, ataxia, confusion, hallucinations, tremor. − Acute neuropathy	Elevated serum toluene levels
Colchicine	− Day 1: nausea, vomiting, hypovolemia − Day 1‐7: Multi‐organ failure, neuropathy and encephalopathy − Day 7‐21: Resolution of organ dysfunction, alopecia	
Nitrous oxide	− Dorsal‐column predominant myeloneuropathy − Confusion, paranoia, bizarre behavior	Vitamin B12 levels usually low‐normal, or low (but can be normal) Serum MMA usually elevated
Heroin and other opiates	− Acute heroin‐related polyradiculoneuropathy is identical to GBS − May coexist with CNS sequelae of drug administration	
Acrylamide	− Ascending sensorimotor axonal neuropathy − Autonomic dysfunction − Cerebellar syndrome − Encephalopathy	Usually intentional poisoning
Dioxins	− Ascending, sensory‐predominant neuropathy. − Hepatitis − Typical skin changes (chloracne) after 2‐4 weeks +/‐Encephalopathy: irritability, restlessness, insomnia and stupor	Exposures: occupational or intentional poisoning Serum dioxin levels are diagnostic
Hexane/ Hexacarbons^2^	− Glue sniffers/”huffers” − Visual hallucinations − Distal, ascending, sensorimotor neuropathy	
Methyl bromide	‐Sensorimotor neuropathy +/‐encephalopathy	Exposures: fumigants, refrigeration materials, fire extinguishers
Tin^2^	− Limbic‐cerebellar syndrome − Hyperphagia, thirst, aggressiveness and ataxia. − Hearing impairment − Axonal sensorimotor neuropathy	Exposure: electronics, plastics and soldering industries Urine tin levels peak day 4‐10
Trichlorethylene^2^	− Encephalopathy (dizziness, headache, confusion, coma) − Trigeminal neuroopathy − Diffuse sensorimotor neuropathy	Exposure(occupational): degreasing solvents, flame retardants, cleaning solutions
Carbon disulfide^2^	− Mood swings, psychiatric disturbances, confusion − Extrapyramidal syndromes − Sensorimotor neuropathy	Exposure: occupational; disulfiram overdose
Carbon monoxide^2^	− Headache, confusion, visual change +/‐ polyradiculoneuritis and bilateral facial palsy	Elevated blood carboxyhemoglobin levels
Organophosphates	− Early (hours): cholinergic syndrome, ataxia, hallucinations − Day 1‐4: neuromuscular paralysis − Delayed neuropathic phase: sensorimotor axonal neuropathy	Decreased red blood cell and plasma cholinesterase levels
Scorpion envenomation	− Cholinergic syndrome similar to organophosphate poisoning +/‐Encephalopathy; flaccid quadriparesis	
Mercury	− Gastrointestinal and renal dysfunction − CNS:tremor, delirium, hallucinations, insomnia and agitation − PNS: sensorimotor, length‐dependent polyneuropathy	Exposure: occupational, contaminated seafood, dental amalgam Elevated blood and urine mercury levels
Ciguatoxin^1^	− Encountered in South Pacific − Dysesthetic sensory syndrome: reversal of thermal sensations +/‐ weakness and encephalopathy (rare)	
Tetrodotoxin^1^	− Rapid onset paralytic syndrome within 60 min of ingestion − Encephalopathy exceptionally rare	Exposure: Inadequately prepared puffer fish (Japan)

CNS, Central Nervous System; CO2, carbon dioxide; CSF, cerebrospinal fluid; CT, computed tomography; ELISA, enzyme‐linked immunosorbent assay; EMG, Electromyography; FBC, full blood count; GBS, Guillain‐Barre Syndrome; MMA, Methylmalonic acid; MRI, Magnetic resonance imaging; NCS, nerve conduction studies; PCR, polymerase chain reaction; PET, Positron emission tomography; PNS, Peripheral Nervous System; RBC, red blood cells; RT‐PCR, reverse transcriptase PCR.

^1^ Presentation usually largely dominated by peripheral nervous system involvement. CNS signs rare, but occasionally reported;

^2^ Presentation usually largely dominated by central nervous system involvement. PNS signs rare, but occasionally reported

## Metabolic and Malignancy

### Metabolic

The principal metabolic conditions to rule out in the setting of GBS + CNS are the porphyrias. These inherited disorders, caused by partial enzyme deficiencies in the haem biosynthetic pathway, are extremely rare and most neurologists will go a whole career without encountering a case.

Porphyrias are usually dominantly inherited, though only 10‐20% of gene carriers will ever suffer an acute attack.^29^ Attacks are often precipitated by increased flux through the haem synthesis pathway, such as by pathway enzyme induction (generally by drugs ‐hormonal contraceptives, certain antibiotics and anticonvulsants‐ but also by other factors including low carbohydrate intake and alcohol binging), or increased metabolic demand through infection, or surgery.^29^ Under these circumstances, insufficient enzyme function results in accumulation of toxic haem precursors, and clinical manifestations. Though no sex differences in gene carriage exist, women of reproductive age are five times more likely to experience an acute porphyric attack, probably due to hormonal fluctuations.^29^, ^30^

Porphyric attacks often begin with abdominal pain, at times so severe as to result in exploratory laparotomies (ironically aggravating the attack), followed by the development of neuropsychiatric symptoms. These may be mild (insomnia, agitation) but if unchecked progress to confusion, delirium and seizures.^31^ Up to 40% of affected individuals will develop a combined autonomic and motor‐predominant axonal porphyric neuropathy, starting within 1 month of symptom onset in 80% of cases and sometimes as early as 3 days.^31^ In contrast to GBS, porphyric neuropathy often begins in the arms, an important clinical clue^32^. Autonomic dysfunction is often profound; sinus tachycardia is almost universal, while extreme blood pressure lability is not uncommon. PRES is in fact the most common neuroimaging finding during an attack. Cerebrospinal fluid albuminocytologic dissociation and SIADH‐related hyponatremia (detected in 40% of patients) may further confuse the diagnostic process^31^, ^33^. Urine often darkens upon standing, a fortuitous observation which has provided the clue to diagnosis in some cases.

### Malignancy

One should be particularly cautious about making a diagnosis of GBS in patients with known or suspected malignant disease. Not only are these patients prone to developing weakness through direct nerve infiltration in leptomeningeal metastatic disease, but they are often exposed to vast arrays of potentially neurotoxic chemotherapies as well as frequently suffering nutritional deficiencies.

Leptomeningeal metastases in particular can mimic GBS. These affects 5‐8% of patients with solid tumors (especially adenocarcinomas of the breast and lung, and melanoma), and up to 15% of those with hematologic malignancies.^34^, ^35^ Most affected individuals have a known malignancy, though occasionally it can be the presenting symptom.^35^ Clinical manifestations are protean. Involvement of the spinal nerve roots can produce rapidly progressive weakness, dermatomal sensory loss, radicular pain, and sphincter involvement, while intracranial disease can compromise cranial nerve function.^35^ Symptomatic intracranial hypertension, resulting from impaired CSF resorption often ensues, manifesting as headache, vomiting and altered consciousness.^34^ MRI with contrast, which should be performed prior to lumbar puncture, shows meningeal contrast uptake in the majority of cases though the gold standard for diagnosis remains the detection of malignant cells in spinal fluid, which may require repeated examination of large volumes of CSF or occasionally meningeal biopsy.

Differentiation between leptomeningeal metastases and GBS can be challenging in early disease. Clues to the diagnosis of leptomeningeal metastases include asymmetry of weakness and reflexes, and sphincter involvement. Additionally, though repeated CSF examinations may be required to identify malignant cells, other CSF parameters (opening pressure, protein, cell count, glucose) are almost always abnormal.^34^

Paraneoplastic neurologic syndromes must also be borne in mind. These complex, multifaceted disorders often combine both central and peripheral nervous system (PNS) involvement.^36^ In contrast to leptomeningeal disease, paraneoplastic syndromes usually precede the detection of the primary malignancy.^36^ GBS‐like paraneoplastic syndromes occur mostly in the presence of anti‐Hu antibodies, which is often secondary to small cell lung cancer. The anti‐Hu syndrome generally produces a severe sensory neuronopathy which may mimic sensory variants of GBS, but in 5% mixed motor (often due to a motor neuronopathy) and sensory involvement is the predominant feature;^37^ weakness may be so profound as to require mechanical ventilation. Additionally, patients frequently develop brainstem or limbic encephalitis.^37^ Patients with anti‐CRMP5 antibodies, either alone or in combination with anti‐Hu, may display a similar syndrome.^38^

## Infectious/Immune/Inherited

### Infectious

Several neurotropic (particularly viral) infections have the potential to produce acute flaccid weakness and encephalitis, and are thus important differential diagnoses of GBS + CNS. These agents vary in their mode of transmission, target population, geographic distribution and endemic versus epidemic nature. Important clues to a potential infectious etiology include: (1) recent travel to endemic areas (pathogen specific), (2) fever, rash, meningism, (3) CSF pleocytosis (cell type may give a clue to the etiology) and (4) associated neurological features e.g., extrapyramidal signs in Japanese encephalitis. A brief account of the most notable offenders is given below.

#### Enteroviruses

Though historically poliovirus was the principal infectious agent responsible for acute flaccid myelitis, occasionally combined with polioencephalitis, successful vaccination programs have eradicated this disease from much of the developed world.^39^ Nevertheless, sporadic outbreaks are still occasionally encountered in the developing world and in unvaccinated populations. Outbreaks of other “polio‐like” enteroviruses, predominantly affecting children, and displaying similar tropism for anterior horn cells have emerged in recent years. Examples include enterovirus 71 and enterovirus D68, both causing severe acute flaccid paralysis and brainstem‐predominant encephalitis, sometimes associated with classic hand foot and mouth skin lesions and bulbar or cranial nerve involvement, respectively.^40^, ^41^, ^42^

#### Flaviviruses

A number of arthropod‐borne neuroinvasive flaviviruses, in particular Japanese encephalitis (JEV), West Nile (WNV) and tick‐borne encephalitis (TBE) viruses are well recognized to cause acute flaccid paralysis accompanied by encephalitis.^43^ Generally transmitted to humans through the bite of a mosquito (JEV and WNV) or tick, most infections remain clinically inapparent or produce only mild viral symptoms. In a small proportion however, progression to invasive neurological disease occurs, often with devastating consequences.^43^, ^44^, ^45^, ^46^, ^47^ Flavivirus‐related acute motor syndromes are generally asymmetric and the result of a severe, destructive motor neuronopathy.^46^ Encephalitis characteristically involves brainstem and diencephalic regions, occasionally producing marked thalamic signal abnormalities described as the “double doughnut” sign. This regional predilection for deep gray matter likely explains the frequent extrapyramidal manifestations encountered with several of these disorders.

West Nile virus (WNV) is a mosquito‐borne virus encountered predominantly in the Americas.^46^ West Nile virus encephalitis, predominantly a disease of the elderly or immunocompromised,^46^, ^48^ affects three quarters of symptomatic cases,^46^ and in 20% of individuals is accompanied within 3–7 days of onset by an acute motor neuronopathy, manifesting as acute flaccid paralysis.^49^ A viral exanthem, present in 25% of cases, may be an important clinical clue. Extrapyramidal complications not uncommonly accompany the encephalopathy.^50^

TBE is endemic in Eastern Europe and central Asia. Different subtypes of the virus exist, with varying propensity for producing brainstem‐predominant meningoencephalitis and acute flaccid paralysis.^51^ Both the likelihood of symptomatic infection and of encephalitis increases with age, being most prevalent in those aged over 50 years.^52^

In contrast, Japanese encephalitis, the most common cause of viral encephalitis in Asia, is primarily a disease of the young, with three quarters of those affected being aged under 14 years.^44^ However, immunologically naive travellers of all ages are susceptible to infection, and since most tourists are adults, infections in returning travellers reflect this demographic.^53^, ^54^ JE predominantly manifests with fever, headache and altered level of consciousness; in older individuals, behavioral change, sometimes mistaken for psychiatric disease, may be the sole presenting feature.^44^ Movement disorders, particularly dystonia/parkinsonism frequently develop, as do seizures. Acute flaccid paralysis coexists with encephalitis in 30% of cases, and though generally lower‐limb predominant and asymmetric, can mimic GBS.^44^

#### Lyssaviruses

Rabies is a viral zoonosis acquired through the bite of an infected animal, though 25% of cases have no recollection of animal bites. Rabies is primarily known for its furious form manifesting with hydrophobia, aerophobia, muscle spasms and limbic encephalitis, but 20‐30% of affected individuals present with the paralytic variant, which is thought to result from a combination of anterior horn cell involvement and mixed axonal/demyelinating neuropathy. The neuropathy is possibly immune‐mediated. In its initial stages, paralytic rabies is clinically and electrodiagnostically indistinguishable from GBS.^55^, ^56^ Indeed, GBS is the most common misdiagnosis at onset.

Following inoculation, the virus travels to the central nervous system through retrograde axoplasmic flow. It begins replicating in the dorsal root ganglion, producing viral ganglionitis, often characterized by excruciating pain in the affected limb.^55^, ^56^ Further centripetal spread leads to involvement of the brainstem, hippocampus and hypothalamus, with progressive deterioration in level of consciousness.^55^ Most people seek medical attention within 2 weeks of symptom onset; most will be dead within another 2 weeks. Important clinical clues such as prodromal fever in a patient from a known endemic region, urinary incontinence, percussion myoedema, monomelic onset or single limb predominance, fasciculation in the affected limb, and severe pain may be present, though many exhibit “typical” GBS syndromes.^57^ Cranial nerve and autonomic involvement is common, just as in GBS.^55^

#### Retroviruses

Though the most common peripheral nerve manifestation of HIV infection is a length‐dependent sensory‐predominant neuropathy, GBS‐like presentations can occur, generally as a seroconversion illness or early in the course of infection, before significant immunosuppression has occurred. That being said, HIV‐associated GBS (and its variants such as Miller‐Fisher syndrome) are also reported in late‐stage AIDS patients, as well as during immune reconstitution inflammatory syndromes.^58^, ^59^ HIV‐associated GBS is clinically indistinguishable from “classic” disease, and though conventional teaching dictates that CSF pleocytosis is a common finding, in fact, studies show that the CSF is not infrequently acellular.^60^

Coexisting encephalopathy can occur for multiple reasons, including opportunistic infection (cerebral toxoplasmosis, cryptococcal meningitis, tuberculosis, cytomegalovirus encephalitis), malignancy (CNS lymphoma), progressive multifocal leukoencephalopathy, and HIV‐associated dementia.^61^ In contrast to GBS‐like manifestations however, CNS involvement generally develops in the setting of systemic immunosuppression, meaning that the combination of the two as GBS + CNS is uncommon, but nevertheless important to outrule.

#### Herpesviruses

The most important herpesviruses to consider in GBS + CNS syndromes are cytomegalovirus (CMV) and varicella zoster virus (VZV). Systemic CMV infection rarely occurs outside of extreme immunosuppression as in AIDS and transplant recipients. Retinitis (producing painless visual loss) is the most common presentation, followed by gastrointestinal involvement^62^. Neurological disease occurs in less than 1% of cases.^62^ CMV encephalitis commonly produces lethargy, confusion, cranial nerve palsies, and nystagmus.^62^ Neuroimaging may be normal but CMV PCR on CSF is positive in over 90% of patients. CMV polyradiculopathy, which may mimic GBS, is typified by lower‐limb predominant, excruciatingly painful, ascending flaccid areflexic weakness, and is generally only encountered once CD4 counts drop under 50 cells/mm^3^. In contrast to GBS, sphincter disturbance is common at presentation, and CSF often has a neutrophilic pleocytosis.^62^ Early diagnosis is paramount, as this offers the best hope of survival and functional recovery.

Similarly, VZV can trigger a GBS‐like illness as part of a primary infection, or indeed may produce a meningomyeloradiculitis as part of a reactivation illness.^63^ In both settings, coexistent meningoencephalitis can compromise mental function. In contrast to CMV, immunosuppression is not a prerequisite to developing invasive neurological disease, though advancing age is a risk factor for reactivation.^64^ The typical vesicular rash is a useful clue, but may be absent‐ zoster sine herpete.

#### Bacterial infections

Bacterial infections, *Campylobacter jejuni* in particular, mostly either trigger GBS through immune activation and molecular mimicry, or produce infectious meningitis with involvement of spinal and cranial nerve roots. The list of potential bacterial triggers is vast, has been extensively reviewed,^65^ and is outside the scope of this review. Neuroborreliosis is however worth mentioning, as it commonly manifests with a painful, potentially GBS‐mimicking spinal polyradiculitis, and cranial polyneuritis.^66^ Concomitant Lyme encephalitis is rare (about 5% of cases), and usually a late manifestation, but can resemble psychotic or dementing illnesses.^66^, ^67^ Scrub typhus has similarly been associated with GBS alongside mental status alterations (scrub meningoencepahlitis).^68^ Meningeal infiltration with tuberculosis, cryptococcus and syphilis should always be considered, especially in the immunosuppressed.^69^

### Immune

Vasculitides, with their multisystemic involvement and propensity to masquerade, are important to rule out in GBS + CNS. Though traditional medical teaching dictates that vasculitic neuropathies manifest as mononeuritis multiplex, in fact, a not infrequent presentation is that of symmetric sensorimotor polyneuropathy, at times mimicking GBS. Neuropathy may be the presenting feature of systemic inflammatory disease prior to systemic involvement, and in some cases vasculitides remain confined to the peripheral nerves.^70^, ^71^ CNS manifestations of vasculitis are protean, ranging from confusion, cognitive decline and altered mental status through to neuropsychiatric abnormalities (especially in lupus) and focal neurological signs, often the result of ischemic lesions.

In theory, any vasculitis could cause these complications, but in practice most cases of GBS + CNS result from eosinophilic granulomatosis with polyangiitis or systemic lupus erythematosus.^72^, ^73^, ^74^, ^75^ Clinicians should therefore have a low threshold for serological testing for these disorders, even with “barn door” GBS. Systemic symptoms and signs such as fever, weight loss, purpuric rash, active urinary sediment as well as axonal involvement on nerve conduction studies should alert physicians to the diagnosis. Early nerve biopsy, or biopsy of other involved tissue, in suspect cases may secure the diagnosis and allow initiation of immunosuppressive treatment.

In patients with a subacute neurocognitive syndrome, the autoimmune encephalitides should also be kept in mind. Their syndromic presentations vary according to the associated antibody and the age of the patient, but for the most part comprise combinations of cognitive decline, psychiatric symptoms, movement disorders, seizures, and autonomic dysfunction.^76^, ^77^ If present, signal abnormalities on MRI and CSF pleocytosis are further pointers to the diagnosis.^76^, ^77^ Rarely, autoimmune encephalitides may also have peripheral nerve manifestations mimicking GBS, though this may be overlooked owing to the significant disturbances in mental status. In particular, antibodies targeting glutamate receptors (NMDA and AMPA receptors) have been reported to produce a destructive, generally motor‐predominant neuritis mimicking GBS,^78^ which may precede or develop concurrently with the classic CNS manifestations.^79^ The underlying pathomechanisms remain unclear. Certainly, peripheral nerves are rich in NMDA and AMPA receptors,^80^ opening a number of possibilities, including co‐targeting of central and peripheral epitopes by common antibodies, exposure of peripheral NMDA and AMPA receptors as a result of neuritis with secondary CNS manifestations (akin perhaps to the pathophysiology of post‐HSV anti‐NMDA receptor encephalitis), immune‐mediated motor neuronopathy (glutamate receptors are highly distributed in anterior horn cells) or the presence of other coexisting autoimmune or paraneoplastic antibodies.^78^ Patients with CASPR2 antibodies typically present with limbic encephalitis, Morvan’s syndrome, dysautonomia, and peripheral nerve hyperexcitability, but an association with a syndrome resembling GBS has been recorded^81^.

### Inherited

X‐linked Charcot Marie Tooth disease (CMT 1X) caused by mutations in the *GJB1* gene encoding the gap junction protein, connexin‐32, is the second most common inherited demyelinating neuropathy^82^. CMT 1X is characterized by the insidious onset of a slowly progressive peripheral sensorimotor neuropathy, which generally manifests before the age of 25 years, the phenotype being less severe in women due to X chromosome inactivation. However, superimposed on this chronic neuropathy, patients frequently develop transient neurologic dysfunction triggered by febrile illness, physical exertion or high‐altitude exposure. Episodic symptoms can include hemiparesis, paraparesis or quadriparesis, which are often misdiagnosed as GBS; demyelinating features on electrophysiologic testing can lead to misdiagnosis^83^. Transient CNS dysfunction in CMT 1X can also manifest as ataxia, dysarthria or altered level of consciousness and may be associated with ADEM‐like white matter abnormalities on brain imaging, which can persist^82^.

## Nutritional Syndromes

The principal nutritional disorder to consider in the differential diagnosis of GBS + CNS is thiamine deficiency, producing not only thiamine neuropathy, but also sometimes Wernicke’s encephalopathy, or the more irreversible Korsakoff syndrome.^84^ The classic Wernicke’s triad of confusion, ataxia and ophthalmoplegia can bear striking resemblance to anti‐Gq1b spectrum disorders. Thiamine neuropathy generally produces a symmetric, length‐dependent axonal sensorimotor neuropathy which begins in the lower limbs and progresses centripetally with time, in contrast to GBS which tends to begin with combined proximal and distal weakness.^84^ Dysphagia, vocal cord dysfunction, dependent edema, and elevations in serum lactate are other possible clues to the diagnosis.^85^

Thiamine deficiency may result from insufficient dietary consumption (chronic alcoholism, anorexia nervosa, low thiamine diets, hyperemesis), impaired intestinal uptake (gut failure, surgical resection or diversion procedures and bariatric surgery) or increased metabolic demand e.g., pregnancy and lactation.^84^, ^86^ Approximately 2–4 weeks is required for bodily stores to be depleted and clinical manifestations to arise, though this may be shorter under conditions of physiologic stress e.g., pregnancy. A glucose load in the setting of relative or absolute thiamine deficiency may also precipitate the syndrome.^87^ Though symmetric MRI signal alterations in the thalami, mammillary bodies and periaqueductal regions may be a tip‐off, empiric treatment should be started upon any clinical suspicion, prior to confirmatory testing.^88^

## Drugs and Toxins

A myriad of drugs and toxins can produce rapidly progressive paralysis accompanied by central nervous system dysfunction (Table 5). Patients can be exposed to these through inadvertent environmental exposure, self‐harm attempts, criminal poisoning, or as a known side‐effect of prescribed medications. Most toxic neuropathies produce a length‐dependent sensorimotor axonal neuropathy,^89^ and such a finding on neurophysiologic testing should always lead one to consider this diagnosis.

Certain toxindromes, particularly arsenic and thallium intoxication, characteristically produce a GBS + CNS syndrome. Early symptoms are relatively nonspecific, frequently leading to delays in diagnosis. Indeed it can take 1‐2 weeks for the complete clinical picture to emerge, by which stage valuable time for therapeutic intervention has been lost.

## Arsenic

Acute arsenic poisoning generally results from intentional oral administration, often of rodenticides, as suicide or murder attempts.^89^ Initial symptoms are generally gastrointestinal in nature, including nausea, vomiting, colicky abdominal pain, and characteristically voluminous ‘bloody rice water’ diarrhea.^90^ Vasodilatation, capillary leak and intestinal fluid losses may lead to vasogenic shock. Cardiac arrhythmias and heart failure may develop.^91^ Neuropathy generally appears 7–14 days after ingestion. It begins with distal burning dysesthesia, followed by length‐dependent large and small fiber sensory loss, producing ataxia and weakness in a distal‐to‐proximal fashion, which may mimic GBS.^92^ Paralysis may be so severe as to require mechanical ventilation.^93^ Important clinical clues include the presence of white transverse lines on the nails (Mee’s lines), palmo‐plantar keratosis, hyperpigmentation and desquamation.^90^ Acute psychosis and encephalopathy are also described as part of the toxindrome.^90^, ^91^, ^94^, ^95^

## Thallium

Thallium poisoning, generally encountered following intentional criminal administration, follows a similar pattern to arsenic. Oral ingestion rapidly engenders severe gastrointestinal distress (nausea, vomiting, abdominal pain) followed 2–5 days later by ascending sensory symptoms.^96^, ^97^ Initial severe dysesthesia of the palms and soles are followed by ascending weakness, hours to days after ingestion.^98^ Autonomic involvement is common and cardiovascular compromise may ensue.^98^ Central nervous system involvement comprising psychiatric symptoms, movement disorders and encephalopathy are common.^98^ Optic neuropathies are also frequently described.^96^, ^97^ As in arsenic poisoning, patients can develop Mee’s lines. Dermatitis and stomatitis of the variety encountered in pellagra is often seen, as thallium interferes with riboflavin metabolism. Alopecia, beginning two weeks after ingestion, is characteristic and an important clinical clue.^96^

### Glycol ingestion

Both ethylene and diethylene glycol are found in a number household products including antifreeze, windshield washer fluid and brake fluid. Ingestion produces dose‐dependent toxindromes which develop in phases.^99^ The first phase, beginning within hours, is one of inebriation with ataxia, stupor and occasionally seizures. This is followed by a second phase with renal failure, gap metabolic acidosis and cardiopulmonary compromise.^100^ Finally, often in a delayed fashion 1–2 weeks after ingestion, patients develop flaccid areflexic limb and cranial nerve weakness (often an inflammatory demyelinating polyradiculoneuropathy) possibly as a reaction to deposition of toxic metabolites.^99^, ^100^, ^101^ Bilateral facial nerve involvement is characteristic, bulbar weakness is common and CSF protein is characteristically elevated; CNS signs, including parkinsonism, may be present.^99^ The syndrome is generally only partially reversible.^100^, ^101^

## Environmental toxins

Environmental toxins can also be responsible for GBS + CNS syndromes. One important example is ciguatera poisoning.^102^ This condition, mainly encountered in the South Pacific, is caused by ingestion of reef fish contaminated by ciguatoxin.^102^ The resulting toxindrome is quite specific, producing severe painful paraesthesia which spread centrifugally, as well as characteristic reversal of thermal sensations, whereby hot objects feel cold, and cold objects burning hot. Hot showers and micturition can be particularly distressing. Weakness, either due to direct involvement of motor nerves or polymyositis, and frequent autonomic involvement may lead to an erroneous diagnosis of GBS.^103^ Severe cases may be complicated by delirium, confusion and psychosis.

^104^

## Iatrogenic causes

Drug‐induced peripheral neuropathies account for < 5% of all neuropathies, apart from in the chemotherapy‐treated population, where the rate may be over 50%.^105^ These syndromes generally evolve over weeks to months, and are usually sensory‐predominant with little motor involvement.^105^ Mis‐interpretation as GBS is therefore rare.

One notable exception is that of the newer biologic agents, particularly TNF‐alpha antagonists (infliximab, etanercept, adalimumab), which are frequently employed in the treatment of systemic inflammatory/autoimmune disorders. Administration of these agents has been reported to be associated with GBS and its variants.^105^, ^106^ Interferons have also infrequently been associated with GBS‐like immune neuropathies.^105^ CNS involvement in these scenarios could be the result of the primary disease process, opportunistic infection, or concomitant drug‐related CNS demyelination.^107^

## Conclusion

The above narrative review details a systematic, step‐by‐step approach to clinical cases of apparent GBS complicated by mental status abnormalities, a syndrome which in our opinion is under‐appreciated in the medical literature. Our discussion is not intended as a systematic review of all possible causes, but rather as a logical framework within which to evaluate such patients. While GBS itself can occasionally produce mental status alterations, this should remain a diagnosis of exclusion. Significant depression in level of consciousness, seizures, movement disorders as well as immunosuppression and at‐risk travel histories should prompt exhaustive searches for alternative explanations. Moreover, the medical interview, combined with a high degree of suspicion is often key to identifying various toxindromes, the full clinical pictures of which may take weeks to develop. Many GBS + CNS disorders are treatable, with patient outcomes being critically dependent on early recognition. A structured approach such as that presented here is therefore paramount.

## Author Contributions

1) conception and design of the study, 2) acquisition and analysis of data. 3) drafting a significant portion of the manuscript or figures. Eoin Mulroy 1, 2, 3. Neil E Anderson 1, 2, 3

## Conflicts of interest

Nothing to report.
